# Bacterial infections in patients hospitalized with COVID-19

**DOI:** 10.1007/s11739-021-02824-7

**Published:** 2021-08-18

**Authors:** Víctor Moreno-Torres, Carmen de Mendoza, Sara de la Fuente, Enrique Sánchez, María Martínez-Urbistondo, Jesús Herráiz, Andrea Gutiérrez, Ángela Gutiérrez, Carlos Hernández, Alejandro Callejas, Carmen Maínez, Ana Royuela, Valentín Cuervas-Mons, the Puerta de Hierro COVID-19 working group, A. Fernández-Cruz, E. Múñez, R. Malo de Molina, I. Pintos, A. Díaz de Santiago, A. Ramos, P. Mills, P. Laguna, G. Vázquez, M. Valle, A. Muñoz, B. Cantos, J. Calderón, A Ángel-Moreno, I. Baños, E Montero, M. C. Carreño, Y. Romero, R. Muñoz, P. Durán, S. Mellor-Pita, P. Tutor, M. Aguilar, G. Díaz, C. García, B. Jara, R. Laporta, M. T. Lázaro, C. López, P. Mínguez, A. Trisán, R. Carabias, M. Erro, B. Agudo, J. Aller, R. Benlloch, M. R. Blasco, M. A. Brito, V. Calvo, M. Calvo, J. Campos, R. Cazorla, M. Cea, H. Cembrero, E. Colino, S. Córda, S. Cruz, G. Del Pozo, C. Del Pozo, M. Elosua, M. Espinosa, C. Fernández, C. Ferre, M. García-Espantaleón, E. A. García-Izquierdo, B. Gil, P. Gómez-Porro, S. González, I. González, G. Escalera, A. I. López, A. Losa, M. E. Marín, I. Martínez, M. E. Martínez, C. Maximiano, M. Méndez, S. Mingo, C. Mitroi, B. Núñez, P. Ortega, J. F. Oteo, N. Pérez, L. Prieto, L. Relea, G. Rodríguez, J. Sabín, J. Sáenz, A. Sánchez, A. Sánchez, J. Sanz, J. Segovia, L. Silva, J. Toquero, M. E. Velasco, S. Villaverde, A. Andrés, S. Blanco, I. Diego, I. Donate, G. Escudero, E. Expósito, A. Galán, S. García, J. Gómez, A. Gutiérrez, V. Edith, I Gutiérrez, F. Martínez, A. Mora, I. Morrás, A. Muñoz, A. Valencia, J. M. Vázquez, A. Arias, J. Bilbao, A. M. Duca, M. A. García-Viejo, J. M. Palau, A. Roldán, R. Castejón, M. J. Citores, S. Rosado, J. A. Vargas, P Ussetti

**Affiliations:** 1 Internal Medicine Department, Hospital Universitario Puerta de Hierro, Majadahonda, Spain; 2 CEU-San Pablo, University, Madrid, Spain; 3 Clinical Biostatistics Unit, Health Research Institute Puerta de Hierro-Segovia de Arana, CIBERESP, Madrid, Spain; 4 Pharmacy Department, Hospital Universitario Puerta de Hierro-Majadahonda, Madrid, Spain; 5 Universidad Autónoma de Madrid, Madrid, Spain

**Keywords:** COVID-19 pneumonia, Bacterial infections, Steroids

## Abstract

Bacterial infections may complicate the course of COVID-19 patients. The rate and predictors of bacterial infections were examined in patients consecutively admitted with COVID-19 at one tertiary hospital in Madrid between March 1st and April 30th, 2020. Among 1594 hospitalized patients with COVID-19, 135 (8.5%) experienced bacterial infectious events, distributed as follows: urinary tract infections (32.6%), bacteremia (31.9%), pneumonia (31.8%), intra-abdominal infections (6.7%) and skin and soft tissue infections (6.7%). Independent predictors of bacterial infections were older age, neurological disease, prior immunosuppression and ICU admission (*p* < 0.05). Patients with bacterial infections who more frequently received steroids and tocilizumab, progressed to lower Sap02/FiO2 ratios, and experienced more severe ARDS (*p* < 0.001). The mortality rate was significantly higher in patients with bacterial infections as compared to the rest (25% vs 6.7%, respectively; *p* < 0.001). In multivariate analyses, older age, prior neurological or kidney disease, immunosuppression and ARDS severity were associated with an increased mortality (*p* < 0.05) while bacterial infections were not. Conversely, the use of steroids or steroids plus tocilizumab did not confer a higher risk of bacterial infections and improved survival rates. Bacterial infections occurred in 8.5% of patients hospitalized with COVID-19 during the first wave of the pandemic. They were not independently associated with increased mortality rates. Baseline COVID-19 severity rather than the incidence of bacterial infections seems to contribute to mortality. When indicated, the use of steroids or steroids plus tocilizumab might improve survival in this population.

## Introduction

Already in the middle of 2021, the SARS-CoV-2 infection continues, being the largest health problem worldwide [1]. Since its first outbreak in December 2019 and the official consideration as a pandemic by the WHO, the disease has spread through the world affecting practically every community. COVID-19 disease occurs in several phases in which some patients require hospitalization due to acute respiratory distress (ARDS) after the so-called cytokine storm or cytokine release syndrome [2]. Among different therapeutic options, treatment with corticosteroids and tocilizumab has been widely used with conflicting results for the latter [3–5]. In addition, the use of these immunosuppressants could increase the risk of secondary infections [6, 7]; not to forget that respiratory viral infections may also predispose to bacterial [8].

In the present study, our objective was to describe and analyze the prevalence of bacterial infections and the main risk factors for infections, other than SARS-CoV-2, in patients admitted due to COVID-19 pneumonia during the first period of the pandemic. We analyzed the rate of patients with bacterial infections as well as their impact on OVID-19 morbidity and mortality. Knowing the characteristics of bacterial infections in patients with COVID-19 could help us optimize therapeutic options, and corticosteroids and/or antibiotherapy use in patients at risk.

## Patients and methods

### Study design and patients

This retrospective observational cohort study was performed at Hospital Puerta de Hierro-Majadahonda, a large tertiary university hospital located in Madrid, one of the most affected regions by COVID-19 during the first wave. The study population consisted of adult patients who were admitted because of interstitial pneumonia due to suspected or confirmed SARS-CoV-2 between March 1st and April 30th, coinciding with lockdown and the first pandemic wave. According to this, both RT-PCR confirmed SARS-CoV-2 infection and suspected SARS-CoV-2 interstitial pneumonia (in the absence of other causes) were included. Follow-up continued to June 30th, 2020. The study was performed in accordance with the ethical standards as laid down in the 1964 Declaration of Helsinki and was approved by the hospital’s Research Ethics Commission. All patients were requested their consent to register their clinical information into a database for epidemiological studies.

### Local treatment protocol

During the first pandemic wave, immunosuppressive and antibiotic therapy was protocolized in our center. All patients with interstitial pneumonia received azithromycin and hydroxicloroquine during 3 and 5 days, respectively. Lopinavir/ritonavir was used if the patient presented hypoxemia during the first 10 days after symptom onset while interferon-beta (IFN-β) use was determined by the presence of respiratory insufficiency. Steroids and/or tocilizumab were considered in case of ARDS 7 days after symptom onset and in the absence of data suggestive of bacterial superinfection. Empirical antibiotic therapy with cephalosporins in addition to azithromycin relied on the physician’s criteria for each situation.

### Data collection and outcomes

Electronic medical records for all hospital admitted patients with COVID-19 pneumonia were reviewed. The main demographics, the baseline comorbidities including immunosuppression and immunosuppressive treatment, microbiological tests (respiratory samples, urinary antigen test, blood, urine and other sites cultures depending on the foci), immunosuppressive treatment received to treat COVID-19 ARDS and outcomes, were collected directly from the electronic medical records. All data were registered by a primary reviewer and subsequently checked by at least two senior physicians.

### Definitions

Immunosuppression was defined either as the presence of hematological disease (active lymphoproliferative, myeloproliferative disorders or bone marrow transplantation), solid organ transplantation, active and disseminated solid organ neoplasm or any condition, including autoimmune disease (e.g., Systemic Lupus Erythematosus, Sjögren Syndrome or Inflammatory Bowel Disease…) that had required immunosuppressive treatment for at least 3 months. Immunosuppressive treatment was considered when the patient was either receiving active treatment at the time of admission, including equivalent doses of prednisone above 5 mg, or had received chemotherapy or immunotherapy 6 months before disease onset.

Acute Respiratory Distress Syndrome (ARDS) and its severity were defined according to the Berlin definition [9]. In patients whose partial pressure of oxygen (PaO2) was unavailable; SapO2/FiO2 ratio was used to assess ARDS and severity [10]. Mild ARDS was considered when PaO2/FiO2 ratio was > 200 mmHg or SapO2/FiO2 > 235 mmHg, moderate when PaO2/FiO2 ratio was > 100 mmHg or SapO2/FiO2 > 160 mmHg and severe when PaO2/FiO2 ratio was ≤ 100 mmHg.

Bacterial infection was documented as the presence of one of the following: fever or chills in the absence of other etiologies, purulent sputum, catheter swelling, inflammatory diarrhea or abdominal pain, along with microbiologic results including blood and urine cultures, upper and lower respiratory samples, cerebrospinal fluid, urinary antigen tests, intraoperative samples, glutamate dehydrogenase test or Clostridioides toxin in stool test. Bacterial pathogen evaluation in blood, fluids, sputum and other samples was performed according to standard microbiological procedures during hospital admission. In addition, laboratory parameters (neutrophilia or procalcitonin elevation) along with radiological and intraoperative findings were considered, particularly in those patients whose microbiological confirmation was not possible. Every suspected infection and its pathogen were carefully and individually assessed by two senior physicians to determine clinical relevance and to avoid selection bias.

Microorganisms were considered multidrug resistant (MDR) if they were resistant to one or more agents in at least three antibiotic classes (beta-lactams, fluoroquinolones, macrolides, aminoglycosides or sulfamides), while difficult-to-treat resistance (DTR) was defined by the resistance to all first-line agents, including all beta-lactams and fluoroquinolones [11, 12].

### Statistical analysis

Descriptive analyses were performed through the mean (standard deviation, SD) for quantitative variables and absolute (and relative) frequencies for the categorical. An univariate analysis was performed comparing those characteristics for patients who suffered bacterial infections vs those who did not, and also between survivors and non-survivors by means of chi-square test in case of categorical variables and Mann–Whitney’s *U* or Student *t*-test for numerical variables depending on their distributions and performing the Levène test. Potential confounding variables were entered into two multivariable logistic regression analyses. The objective was to identify factors related with the risk of bacterial infection and mortality, respectively. For all analyses, significance level was defined as a *p* value below 0.05. Statistical analysis was performed using SPSS software version 26.0 (IBM, Spain).

## Results

### Patients characteristics

A total of 1594 patients admitted because of suspected or confirmed SARS-CoV-2 pneumonia between March and April 2020 were analyzed. Mean age was 65 years old, 62.1% were male and 87.2% had a positive PCR for SARS-CoV-2 at the time of admission. Overall, 135 patients (8.5%) developed a bacterial infection during admission (Table 1). Patients with infections were significantly older (mean age 68 vs 64.5, *p* = 0.007) and presented higher rates of baseline comorbidities as diabetes (27.4% vs 16.7%, *p* = 0.002), neurological disease (20.7% vs 13.5%, *p* = 0.021) and kidney disease (13.3% vs 6.4%, *p* = 0.021). In addition, 28.9% of patients with bacterial infection were immunosuppressed (vs 8.7%, *p* < 0.0001), being the main causes: autoimmune disease (8.9%), hematological disease (7.4%), solid organ neoplasm (5.9%) and solid organ transplantation (4.4%). As a result, 25.2% of infected individuals were receiving immunosuppressive treatment.

**Table 1:** Baseline characteristics of the study population

	Total *N* (%)	Bacterial infections		*p* value*
		Yes *n* (%)	No *n* (%)	
COVID-19 hospitalized patients	1594	135	1459	–
Mean age (mean, SD)	65 (15.0)	68 (14.3)	64.5 (14.9)	0.007
Male sex	990 (62.1)	87 (64.4)	903 (61.9)	Ns
High blood pressure	699 (43.9)	65 (48.1)	634 (43.5)	Ns
Diabetes mellitus	281 (17.6)	37 (27.4)	244 (16.7)	0.002
Obesity	424 (26.6)	36 (30)	388 (35.7)	Ns
Heart disease	270 (16.9)	28 (20.7)	242 (16.6)	Ns
Neurological disease	225 (14.1)	28 (20.7)	197 (13.5)	0.021
Lung disease	248 (15.6)	26 (19.3)	222 (15.2)	Ns
Kidney disease	112 (7)	18 (13.3)	94 (6.4)	0.003
Liver disease	48 (3)	6 (4.4)	42 (2.9)	Ns
Immunosuppression	166 (10.4)	39 (28.9)	127 (8.7)	< 0.0001
Autoimmune disease	65 (4.1)	12 (8.9)	53 (3.6)	0.01
Solid organ transplantation	30 (1.9)	6 (4.4)	24 (1.6)	0.036
Hematological disease	35 (2.2)	10 (7.4)	25 (1.7)	0.000
Solid organ neoplasm	32 (2)	8 (5.9)	24 (1.6)	0.004
Others	4 (0.25)	–	–	–
Immunosuppressive treatment	139 (8.7)	34 (25.2)	105 (7.2)	0.000
Steroids	68 (4.3)	15 (11.1)	53 (3.6)	0.000
Calcineurin inhibitors	25 (1.6)	6 (4.4)	19 (1.3)	0.005
Mycophenolate	25 (1.6)	8 (5.9)	17 (1.2)	0.000
Biologicals	30 (1.9)	6 (4.4)	24 (1.6)	0.022
Chemotherapy	16 (1)	6 (4.4)	10 (0.7)	0.000
Others	6 (0.4)	–	–	–

*SD* Standard deviation, *NS* Non-significant

A total of 156 bacterial infections occurred in 135 patients, with significant microbiological isolation in 91.9% of them (Table 2). The main sites of infection were urinary tract (32.6%), lung (31.8%) and bacteremia (31.9%), related to catheter in 67.4% of them. In addition, nine patients (6.7%) presented intra-abdominal or skin and soft tissue infection. Other foci were meningitis, endocarditis, otorhinolaryngologycal site, tuberculosis or septic shock (5.9%).

**Table 2:** Bacterial infections in 135 COVID-19 patients. Anatomic site and microorganism

	*N* (%)
Site of infection	
Lung	43 (31.8)
Community-acquiredy/superinfection	22/43 (51.2)
Nosocomial	21/43 (48.8)
Bacteremia	43 (31.9)
Catheter related	29/43 (67.4)
Primary	14/43 (32.6)
Urinary tract	44 (32.6)
Intra-abdominal	9 (6.7)
Skin and soft tissue	9 (6.7)
Others*	8 (5.9)
Microorganism	
Gram-positive cocci	73 (54.1)
MRSA	11 (8.2)
MSSA	2 (1.5)
CNS	32 (23.7)
Enterococci	34 (25.2)
Streptococci	16 (11.9)
Enterobacterales	40 (29.6)
* E. Coli*	30 (22.2)
* Klebsiella* spp.	16 (11.9)
* Enterobacter s*pp.	3 (2.2)
Others	2 (1.5)
Non-fermentative gram-negative	13 (9.6)
*P. aeruginosa*	12 (8.9)
*Stenotrophomonas maltophilia*	4 (3)
*Acinetobacter* spp.	1 (1)
*Achromobacter* spp.	1 (1)
Anaerobic bacteria	9 (6.7)
* Clostridium difficile*	4 (3)

*MRSA* Methicillin-resistant *Staphylococcus aureus*, *MSSA* Methicillin-sensitive *Staphylococcus aureus*, *CNS* Coagulase-Negative Staphylococci

*Included meningitis (2 cases), endocarditis (2 cases), otorhinolaryngology site (2 cases), tuberculosis (1 case), or septic shock from unknown foci (1 case)

Regarding the observed microorganisms, gram-positive cocci were the most frequent isolation (54.1%). On the other hand, gram-negative bacteria were documented in 40 patients (29.6%) while non-fermentative bacteria were identified in 13 patients (9.6%). Species are also shown in Table 2.

MDR were isolated in 26 patients (19.3%); due to *E. Coli* spp (30.8%), *Pseudomonas* (15.4%), resistant*-staphylococci* (15.4%), *Stenotrophomonas* (15.4%), *Enterobacter* (7.7%), *Klebsiella* (7.7%), *Achromobacter* (3.8%) and *Acinetobacter* species (3.8%). 63.3% of MDR infections happened in ICU admitted patients. In parallel, Gram-negative-DTR were identified in 18 (13.3%), being *E. Coli (*27.8%*), Stenotrophomonas* (22.2%)*, Pseudomonas (*16.7%*), Enterobacter* (11.1%), *Klebsiella* (11.1%), *Achromobacter* (5.5%) and *Acinetobacter* species (5.5%). 77.8% of these isolates were observed in patients admitted to the ICU.

11 patients had no isolate. Foci were: nosocomial pneumonia (four patients), skin and soft tissue (three patients), urinary tract (two patients), diverticulitis (one patient) and septic shock in an immunosuppressed patient with hematological disease.

### Disease severity, treatment and outcomes.

Overall, 90.4% of patients with any bacterial infection have had ARDS in the context of COVID-19 disease vs 74.8% of patients without infectious complications (*p* < 0.001). In addition, these patients had lower SapO2/FiO2 ratios (198 vs 280, *p* < 0.001). Consequently, the patients with bacterial infections had more severe ARDS (40.7% vs 5.1%, *p* < 0.001) when compared with the rest. The immunosuppressive treatment used to treat ARSD was also analyzed. Patients with infections had received more steroids (76.1% vs 56.5%, *p* < 0.0001) and more tocilizumab (40% vs 16.9%, *p* < 0.001).

Considering outcomes, patients who had suffered bacterial infection had longer hospital stay (19.5 vs 8.4 days, *p* < 0.001), had been more frequently admitted to the ICU (40% vs 3.8%, *p* < 0.001) and ICU stays had been significantly higher (27.2 vs 10.4 days, *p* < 0.001). In addition, these patients had higher readmission rates after discharge (14.2% vs 4.9%, *p* < 0.001), 57.9% of them motivated by bacterial infections acquired during first admission. Overall mortality was 15.1%, being significantly higher in patients with bacterial infection (25.03% vs 6.70%, *p* < 0.001).

### Risk factors for bacterial infection

To identify the risk factors associated with bacterial infections in the context of COVID-19, a multivariable analysis was performed considering the patient's baseline characteristics, previous treatments, disease severity and the treatment used for ARDS (Table 3). Independent factors related with bacterial infection were: age (OR 1.02, 95% CI 1.01–1.04, *p* = 0.009), neurological disease (OR 1.69, 95% CI 1.01–2.82 (*p* = 0.046)), immunosuppression (OR 4.41, 95% CI 2.76–7.06, *p* < 0.001) and ICU admission (OR 21.36, 95% CI 13.21–34.55, *p* < 0.001). Neither steroid nor tocilizumab combined with steroid treatment for ARDS were significantly associated with a higher risk of infection after variable adjustment.

**Table 3:** Risk factors for bacterial infections in COVID-19 hospitalized patients

	Univariate analysis*		Multivariate analysis**	
	OR (95% CI)	*p* value	OR (95% CI)	*p* value
Baseline conditions				
Age	1.02 (1.01–1.03)	**0.007**	**1.02 (1.01–1.04)**	**0.009**
DM	1.88 (1.26–2.81)	**0.002**	1.60 (0.99–2.49)	0.059
Neurological disease	1.68 (1.08–2.60)	**0.022**	**1.69 (1.01–2.82)**	**0.046**
Kidney disease	2.23 (1.30–3.82)	**0.003**	1.221 (0.64–2.29)	0.555
Active neoplasm	2.66 (1.52–4.65)	**0.001**	1.047 (0.50–2.20)	0.903
Immunosuppression	4.26 (2.82–6.45)	** < 0.001**	**4.41 (2.76–7.06)**	** < 0.001**
Outcome				
ARDS	3.17 (1.77–5.68)	** < 0.001**	1.34 (0.70–2.55)	0.375
ICU admission	16.69 (10.80–25.81)	** < 0.001**	**21.36 (13.21–34.55)**	** < 0.001**
Treatment				
Steroids	2.46 (1.63–3.70)	** < 0.001**	0.94 (0.55–1.61)	0.828
Tocilizumab	3.29 (2.27–4.76)	** < 0.001**	0.66 (0.12–3.75)	0.636
Steroids* + Tocilizumab^T^	2.43 (1.66–3.58)	** < 0.001**	1.31 (0.80–2.16)	0.282

Ods ratio, confidence intervals and p-values marked with bold indicate statistically significance

*OR* Odds ratio, *CI* Confidence interval, *DM* Diabetes mellitus, *ARDS* Acute respiratory distress syndrome, *ICU* Intensive care Unit

^T^: Product term between steroids and tocilizumab treatment

### Mortality risk factors

**Fig. 1:**
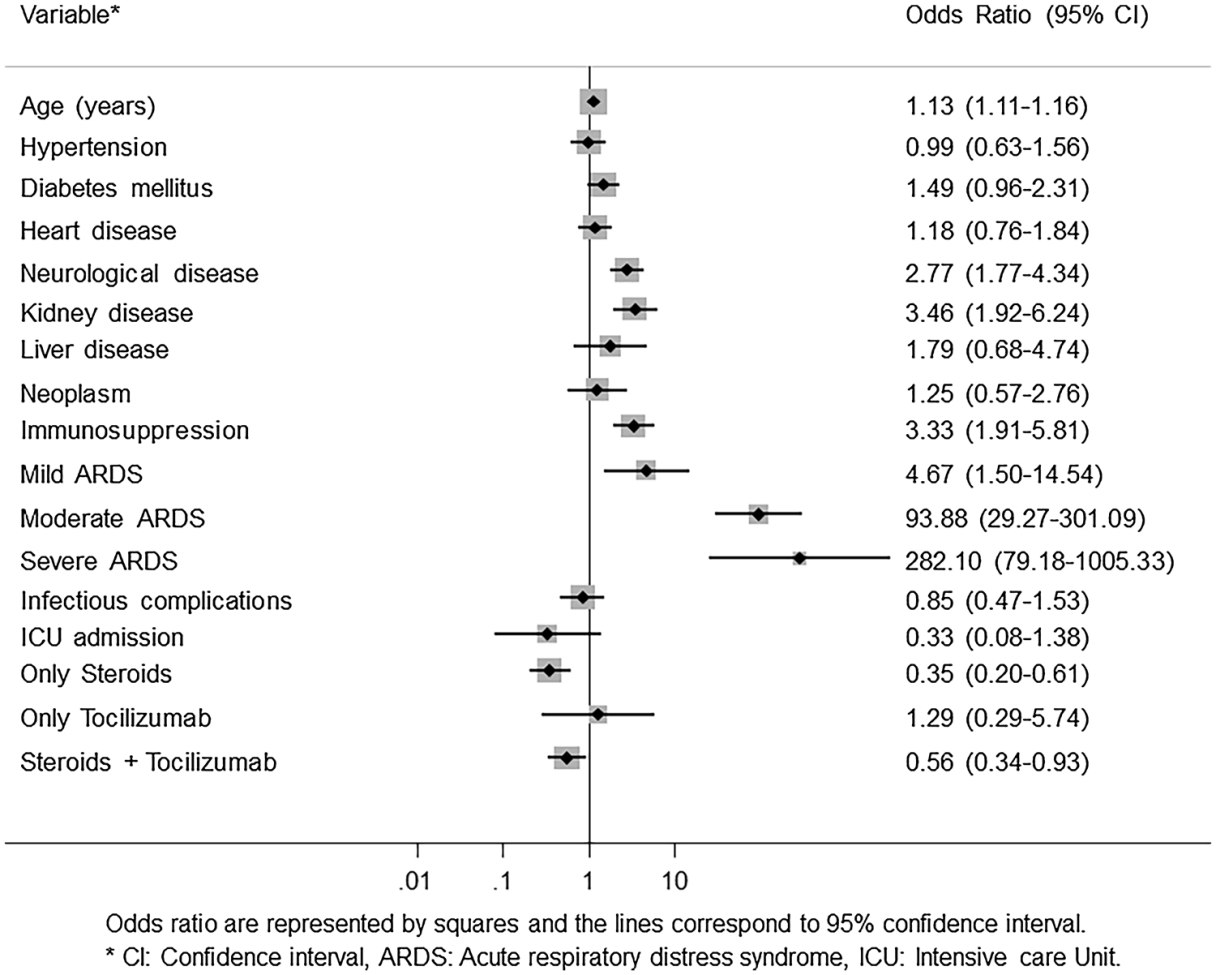
Predictors of mortality in COVID-19 hospitalized patients. Odds ratio are represented by squares and the lines correspond to 95% confidence interval.* CI* Confidence interval,* ARDS* Acute respiratory distress syndrome,* ICU* Intensive care Unit

Since a higher proportion of individuals with bacterial infection died during admission in the univariate analysis, a multivariable analysis was performed to identify mortality risk factors (Fig. 1). Mortality was determined by baseline comorbidities, including age (OR 1.13, 95% CI1.10–1.16, *p* < 0.0001), neurological disease (OR 2.77, 95% CI 1.77–4.34, *p* < 0.0001), kidney disease (OR 3.46, 95% CI 1.92–6.24, *p* < 0.0001), previous immunosuppression (OR 3.33, 95% CI 1.91–5.81, *p* < 0.0001) and by the presence and severity of ARDS: mild ARDS (OR 4.67, 95% CI 1.50–14.54, *p* = 0.008), moderate ARDS (OR 93.88, 95% CI 29.27–301.08, *p* < 0.0001) and severe ARDS (OR 282.10, 95% CI 79.18–1005.33), *p* < 0.0001). By contrast, bacterial infections were not independently associated with mortality (OR 0.85, CI 0.47–1.53, p > 0.05). Steroid treatment (OR 0.35, 95% CI 0.20–0.60, *p* < 0.0001) and the combination of steroids with tocilizumab (OR 0.56, 95% CI 0.34–0.93, *p* < 0.024) showed lower mortality rates.

## Discussion

In this study, we aimed to describe and analyze the burden and risk factors of bacterial infections in patients with COVID-9 disease, since the main therapeutic approaches to date are corticosteroids and tocilizumab, both with recognized potential to develop infections.

We documented a prevalence of 8.5% of bacterial infections, a slightly higher proportion compared to nosocomial and health-care associated infection rates before the pandemic [13, 14]. However, our results are similar to those reported in other COVID-19 cohorts [15, 16]. In addition, we also confirmed that infections are not only caused by respiratory superinfection. There is an important rate of primary and catheter-related bacteremias, urinary tract and abdominal infections, with a significant rate of gram-positive cocci, enterobacterial, non-fermentative and multiresistant pathogens [14, 17, 18]. These are not surprising data since COVID-19 actually can result in long hospital stays, ICU admission, vascular and respiratory devices, malnutrition and a wider use of empirical antibiotherapy, all well-known risk factors for nosocomial infection [19, 20]. Furthermore, SARS-CoV-2 infection could result in a systemic hyperinflammatory disease that carries a state of immunosuppression beyond lung injury [21, 22], contributing to a significant number of infectious complications affecting other sites.

Our data show that, in addition to age and baseline comorbidities, ICU admission was the main risk factor for the development of bacterial infection. At the same time, ARDS severity and the respiratory worsening of patients during the disease mainly determined ICU admission. Other authors have described significantly higher rates of secondary infections in critically ill patients with severe ARDS [23–25]. Moreover, Bardi et al. identified disease severity as the only factor associated with the development of infection in the ICU [26]. The pandemic situation of the first wave implied a lack of material and staff, infrastructural changes and a significant work overload that altered the normal organization of the ICU. As a result, standards of prophylactic care and aseptic conditions of the procedures were difficult to fulfill as usual, justifying the higher rates of infectious complications.

On the other hand, steroid treatment was initially criticized during the first months of the pandemic since there were no robust evidence to support its use. Several reports informed that early treatment with steroids might extend SARS-CoV-2 RNA replication [27, 28]. In addition, bacterial and opportunistic infections during or after steroid treatment are a major concern and recognized side effect, even at low doses and short courses [6, 29, 30], being a possible limitation for its use in COVID-19 patients. As a matter of fact, Obata et al. found that steroids but not tocilizumab were associated with higher rates of bacterial and fungal infection in COVID-19 hospitalized patients [31]. However, in our cohort neither steroid treatment nor tocilizumab exposure carried a significant risk of bacterial infection when adjusted in the multivariate analysis. Consequently, our study confirms the benefit of the anti-inflammatory effect of steroids in COVID-19, overcoming the potential risk of infections in this scenario [3, 32, 33].

In parallel, similar concerns have affected the use of tocilizumab in autoimmune diseases [7, 34], revealing an even higher risk of bacterial infections with tocilizumab than with tumor necrosis factor inhibitors (TNFi) [35]. In our cohort, tocilizumab use was limited (one or two doses), therefore not conditioning the maintained blockage of IL-6R and avoiding the possible long-term immunosuppression and risk of infection [34]. To note, Stone et al. documented fewer serious infections in patients treated with tocilizumab [4] and the recent trial from Veiga et al. showed no differences in the secondary infection rates when tocilizumab was compared with standard care [5]

Finally, the main related mortality factors were again age, comorbidities and ARDS, while bacterial infections were not an independent factor, reinforcing the hypothesis that infections are a surrogate marker of the most fragile or most severely affected patients with COVID-19. Furthermore, steroid and the combination of steroid with tocilizumab provided a protective effect, supporting its role in COVID-19, since they did not lead to more bacterial infections.

According to our findings, questions about antibiotic therapy during COVID-19 disease arise again. Others have observed that, to date, there is no evidence enough to support empirical antimicrobial due to data absence [36]. In parallel, our results support that antibiotic prescription should not be generalized and might be carefully evaluated; above all if we consider that infections are the consequence of severe disease. Consequently, the best approach seems to be ARDS treatment with clinical and microbiological surveillance; waiting to microbiological definite diagnosis rather than early empirical prescription when suspected.

The main limitation of our study was the absence of data regarding the empirical and targeted antibiotic treatment in the cohort. We were not able to understand their role in the risk and courses of bacterial infections. Unfortunately, and despite that treatment during this period of the pandemic was carefully standardized; no information, conclusions or recommendations could be elucidated in this regard.

In summary, nearly 9% of individuals hospitalized with COVID-19 developed bacterial infection during the first pandemic wave. Although this population exhibited an unfavorable clinical profile, bacterial infections were not independently associated with increased mortality rates or with steroid and tocilizumab treatment for ARDS. This result suggests that bacterial infections reflect disease severity rather than contribute to mortality. Immunosuppressive treatment should be used when indicated given that it did not implied higher bacterial infection rates and resulted beneficial for patients with SARS-CoV-2 pneumonia in terms of survival.

## Data Availability

The datasets generated during and/or analyzed during the current study are available from the corresponding author on reasonable request.

## Abbreviations

ARDS: Acute respiratory distress syndrome
DTR: Difficult- to-treat resistance
HBP: High blood pressure
MDR: Multidrug resistant bacteria.
SapO2: Oxygen saturation by pulse oximetry
TNFi: Tumor necrosis factor inhibitors
DM: Diabetes mellitus
FiO2: Fraction of Inspired Oxygen
ICU: Intensive care Unit
PaO2: Partial pressure of oxygen
SOT: Solid organ transplantation

## Code availability

Stata v16 software (StataCorp. 2019. Stata Statistical Software: Release 16. College Station, TX: StataCorp LLC).
